# Screening Estrogenic Activities of Chemicals or Mixtures In Vivo Using Transgenic (*cyp19a1b*-GFP) Zebrafish Embryos

**DOI:** 10.1371/journal.pone.0036069

**Published:** 2012-05-07

**Authors:** François Brion, Yann Le Page, Benjamin Piccini, Olivier Cardoso, Sok-Keng Tong, Bon-chu Chung, Olivier Kah

**Affiliations:** 1 Unité d’Ecotoxicologie in vitro et in vivo, Direction des Risques Chroniques, Institut National de l’Environnement Industriel et des Risques (INERIS), Verneuil-en-Halatte, France; 2 Université de Rennes 1, Institut de Recherche Santé Environnement & Travail (IRSET), INSERM U1085, BIOSIT, Campus de Beaulieu, Rennes France; 3 Taiwan Institute of Molecular Biology, Academia Sinica, Taipei, Taiwan; University of Rouen, France

## Abstract

The tg(*cyp19a1b*-GFP) transgenic zebrafish expresses GFP (green fluorescent protein) under the control of the *cyp19a1b* gene, encoding brain aromatase. This gene has two major characteristics: (i) it is only expressed in radial glial progenitors in the brain of fish and (ii) it is exquisitely sensitive to estrogens. Based on these properties, we demonstrate that natural or synthetic hormones (alone or in binary mixture), including androgens or progestagens, and industrial chemicals induce a concentration-dependent GFP expression in radial glial progenitors. As GFP expression can be quantified by *in vivo* imaging, this model presents a very powerful tool to screen and characterize compounds potentially acting as estrogen mimics either directly or after metabolization by the zebrafish embryo. This study also shows that radial glial cells that act as stem cells are direct targets for a large panel of endocrine disruptors, calling for more attention regarding the impact of environmental estrogens and/or certain pharmaceuticals on brain development. Altogether these data identify this in vivo bioassay as an interesting alternative to detect estrogen mimics in hazard and risk assessment perspective.

## Introduction

Over the last 20 years, numerous examples have documented the adverse reproductive health effects of man-made compounds that, released in the environment, are capable of disrupting the endocrine system in wildlife and human populations [1]. To date, a growing number of structurally and functionally diverse groups of chemicals have been proven or suspected to have endocrine-disrupting chemical (EDCs) activity. Concerns about their effects on human and wildlife reproductive health have stimulated the development and implementation of screening and testing procedures for hazard and risk assessment [2].

EDCs are known to interfere with the endocrine system through multiple signalling pathways. One major mechanism of EDC effects involves their action as estrogen receptors (ERs) agonists. Until now, most studies dedicated to the actions of (xeno)-estrogens have focused on their effects at the level of the gonads and other peripheral tissues [2], [3]. However, there is emerging evidence to show that EDCs, notably (xeno)-estrogens, act in the brain, notably on the development and functioning of the neuroendocrine circuits. However, at the present stage, such potential effects of EDCs are not taken into account in risk assessment, mainly because of the lack of readily accessible and validated models.

In this context, the *cyp19a1b* gene, which encodes a brain form of aromatase (aromatase B) in fish, is of particular relevance for several reasons. First, as documented in different species, this gene exhibits exquisite sensitivity to estrogens [4], [5], [6]. Second, *cyp19a1b* expression is strictly limited to radial glial cells (RGC) that act as neuronal progenitors in both developing and adult fish [7]. Furthermore, several studies point to this gene as a sensitive target for estrogen mimics [8], [9]. We have developed a transgenic zebrafish tg(*cyp19a1b*-GFP) line that expresses GFP under the control of the *cyp19a1b* promoter [10]. As evidenced by careful validation procedures, this line shows perfect co-expression of GFP and endogenous aromatase B in RGC. The reason why *cyp19a1b* is only expressed in radial glial cells (RGC) is not fully understood. Nevertheless, previous studies showed that the estrogenic regulation of *cyp19a1b* expression requires a mandatory interaction between estrogen receptors acting through an estrogen response element (ERE) and an unknown glial factor that binds a sequence located upstream from the ERE in the promoter region of the *cyp19a1b* gene [5]. This results in an intriguing positive auto-regulatory loop through which aromatase, the estrogen-synthesizing enzyme, is up-regulated by E2 (17ß-estradiol). This loop explains why aromatase B expression and activity are so high in the brain of sexually mature adult fish with high levels of sex steroids [11], [12]. In contrast, in embryos, *cyp19a1b* expression is very low but can be strongly activated by E2 exposure as early as 24 hours post-fertilization, i.e. when both estrogen receptors and *cyp19a1b* start to be expressed in the brain [13].

**Figure 1:**
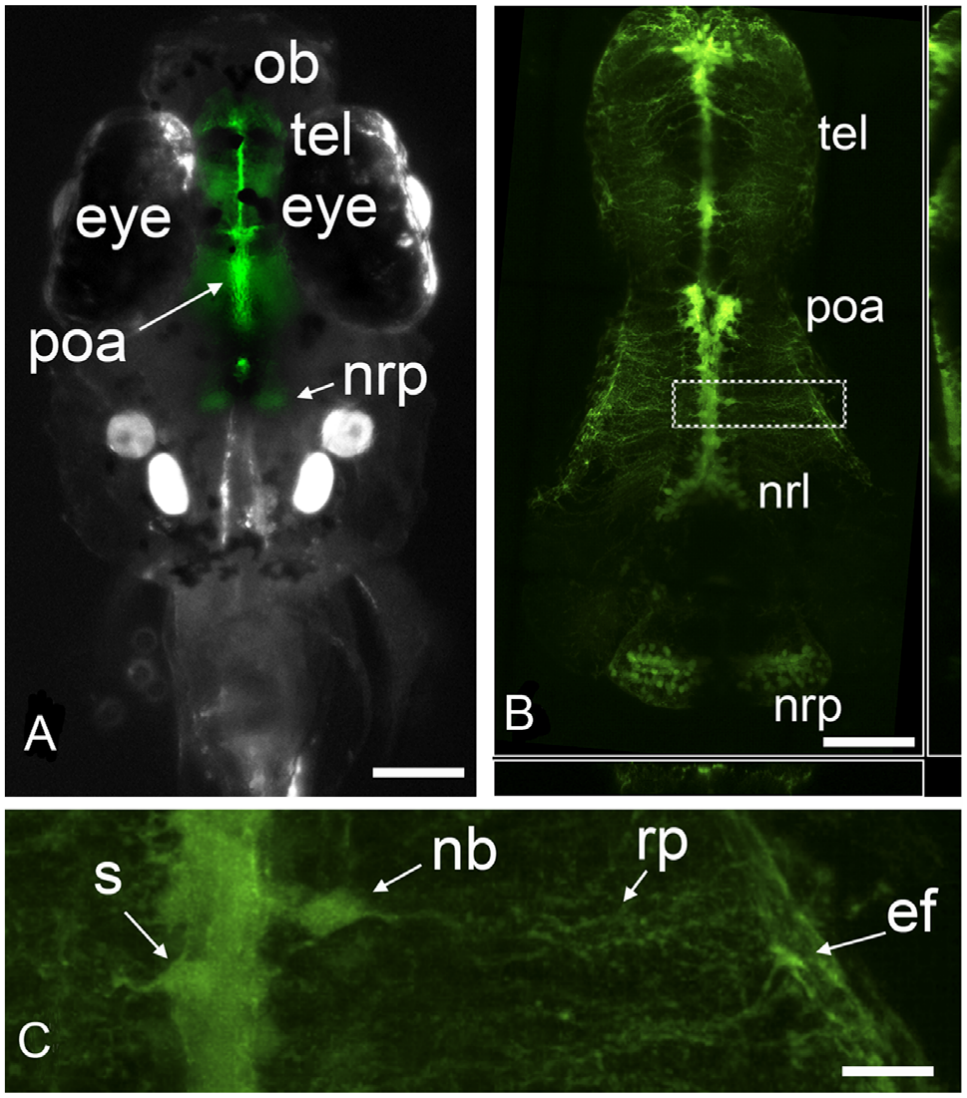
Upon exposure of embryos to estradiol, the tg(*cyp19a1b*-GFP) zebrafish expresses GFP only in radial glial cells. (*a*) Dorsal view of a zebrafish larva treated with 10 nM E2 showing that GFP signal is visible in the brain, notably in the telencephalon (tel), preoptic area (poa), and in the nucleus recessus posterioris (nrp) of the caudal hypothalamus; ob: olfactory bulb. (*b*) High resolution confocal image showing the RGCs in the telencephalon (tel), preoptic area (poa), nucleus recessus lateralis (nrl) and nucleus recessus posterioris (nrp) of the caudal hypothalamus. (c) High power view of the area shown in (*b*). Soma (s) are located along the midline except in the case of newborn cells (nb) undergoing migration (see Figure 2). RGCs have long cytoplasmic radial processes (rp) terminating by end-feet (ef) at the brain surface. (*a*) Bar = 200 µm; (*b*) Bar = 100 µm (c) Bar = 20 µm.

**Table 1:** Calculated Effective concentrations EC_50_ for E2, EE2, E1 and Genistein in transgenic cyp19a1b-GFP zebrafish line based on measurement of either *cyp19a1b* or GFP gene expression by PCR and by image analysis.

Compound	Method	EC_50_ (nM)	±CI 95
E2	*cyp19a1b* mRNA	2.8	2,61−2,99
	GFP mRNA	4.1	1.55−8.16
	Image analysis	0.5	0.45−0.64
EE2	*cyp19a1b* mRNA	0.04	0.031−0.051
	GFP mRNA	0.02	0.012−0.028
	Image analysis	0.01	0.01−0.012
E1	*cyp19a1b* mRNA	2.3	2.14−1.81
	GFP mRNA	1.3	0.88−2.15
	Image analysis	2.4	2.04−2.5
Genistein	*cyp19a1b* mRNA	3545	3052−3556
	GFP mRNA	2466	1172−19261
	Image analysis	2166	1872−2254

*Results are expressed as mean ± confidence interval at 95% (CI 95).*

This study aims at investigating the potential of a large spectrum of ligands, such as natural or synthetic steroids or ubiquitous environmental contaminants, to alter *cyp19a1b*-driven GFP expression in RGCs of developing zebrafish. Because the skull is transparent at these early development stages, GFP expression can be easily imaged and quantified *in vivo* without sacrificing the animals. The main finding of this study is that a number of chemicals can indeed target *cyp19a1b*-GFP expression through ER-activated mechanisms. These chemicals include established (xeno)-estrogens, but also several aromatizable or non-aromatizable androgens and synthetic progestagens, evidencing the usefulness and the validity of the *in vivo* tg(*cyp19a1b*-GFP) zebrafish test for screening compounds, alone or in mixtures.

## Methods

### Ethics

This study was approved by the ethics committees INERIS (Institut National de l’Environnement Industriel et des Risques) and CREEA (Comité Rennais d’Ethique en matière d’Expérimentation Animale) unser permit number EEA B-35-040. All steps have been taken to reduce suffering of animals. Experiments were performed in accordance with European Union regulations concerning the protection of experimental animals (Directive 86/609/EEC).

**Figure 2:**
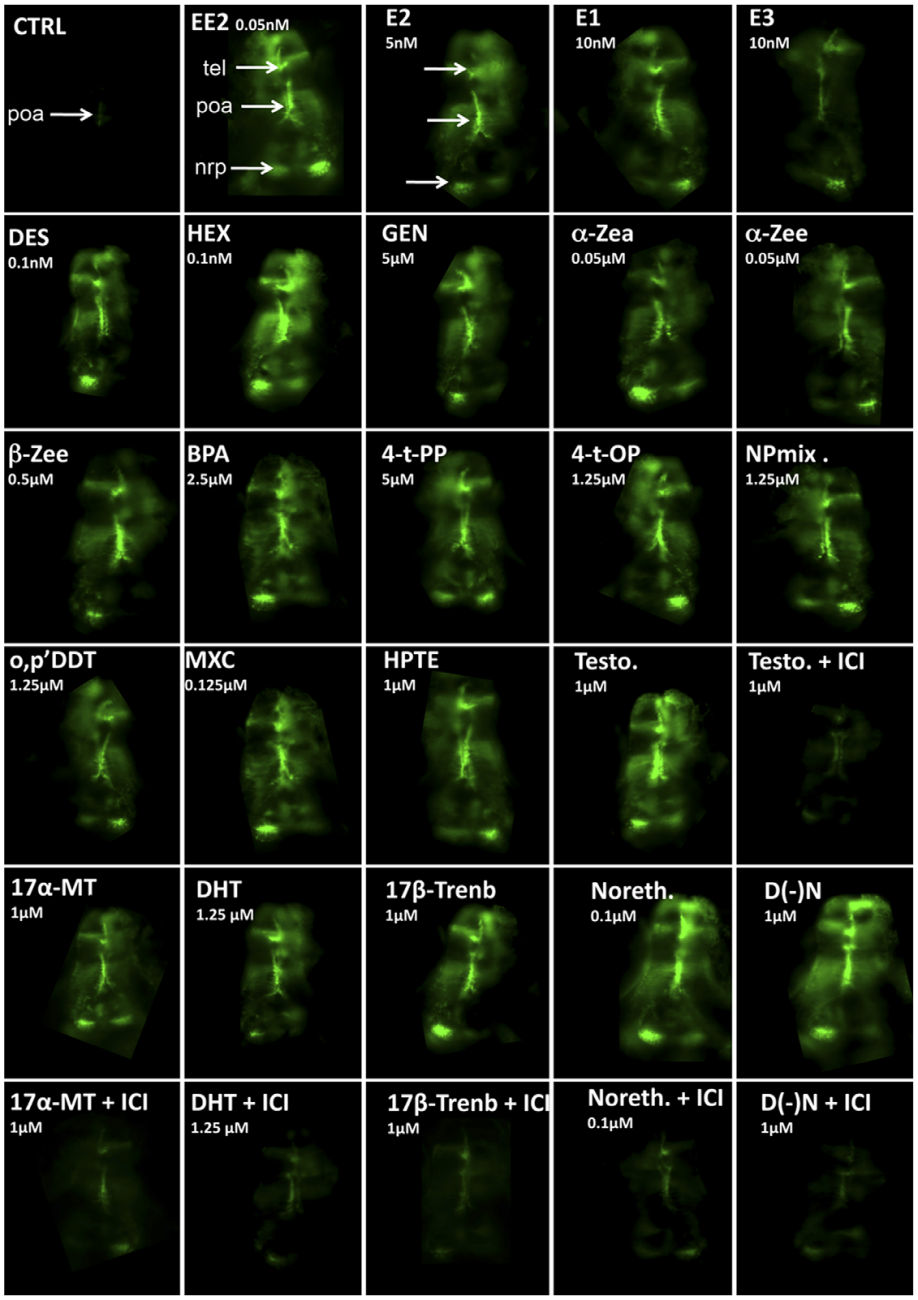
*In vivo* imaging of 5-dpf old live transgenic *cyp19a1b*-GFP zebrafish embryos exposed to chemicals inducing GFP expression in radial glial progenitors. Dorsal views (anterior to the top) of the telencephalon (tel), preoptic area (poa), and nucleus recessus posterioris (nrp) of the caudal hypothalamus. For each chemical the concentration used is indicated. CTRL: solvent control, EE2: 17α-ethinylestradiol, E2: 17β-estradiol, E1: estrone, E3: estriol, DES: diethystilbestrol, HEX: hexestrol, GEN: genistein, α-ZEA: α-zearalenol, α-ZEE: α-zearalanol, β-ZEE: β-zearalanol, BPA: bisphenol A, 4-t-PP: 4-t-pentylphenol, 4-t-OP, 4-t octylphenol, NPmix: mixture of nonylphenol, o,p’DDT: 1,1,1-Trichloro-2-(2-chlorophenyl)-2-(4-chlorophenyl)ethane, MXC: methoxychlor, HPTE 2,2-bis(p-hydroxyphenyl)-1,1,1-trichloroethane, Testo: testosterone, DHT: dihydotestosterone, 17α-MT: 17α-methyltestosterone, 17β-Trenb: 17β-trenbolone, Noreth.: 17α-Ethynyl-19-nortestosterone, D(-)N: 13β-Ethyl-17α-ethynyl-17β-hydroxygon-4-en-3-one, ICI (ICI 182-780).

### Chemicals

17β-estradiol (E2), 17α-ethinylestradiol (EE2), estrone (E1), estriol (E3), diethylstilbestrol (DES), hexestrol (HEX), zearalenol (Zea), α-zearalenol (α-Zee), β-zearalenol (β-Zee) and α-zearalanol (α-Zea), genistein (Gen), diadzein (DZ), 4-tert-octylphenol (4tOP), 4-tert-pentylphenol (4tPP), 4-tert-bisphenol A (BPA), 1,1,1-Trichloro-2-(2-chlorophenyl)-2-(4-chlorophenyl)ethane (o,p’-DDT), Methoxychlor (MXC), 1,1,1-Trichloro-2,2-bis(4-hydroxyphenyl)ethane (HPTE), Chlordecone (Chlo), Endosulfan (Endo), testosterone (Testo), 17α-methyltestosterone (17α-MT), 11-ketotestosterone (11-KT), 4,5α-dihydrotestosterone (DHT), 17β-trenbolone (Trenb), metribolone (R1881), 17α-Ethynyl-19-nortestosterone (norethindrone, NOR), 13β-Ethyl-17α-ethynyl-17β-hydroxygon-4-en-3-one (Levonorgestrel, D(-)N), rifampicine (RIF), dexamethasone (DEX), spironolactone (SPI), corticosterone (COR), Benzophenone (BP), DiBenzo[a]anthracene, (diB[a]A), Benzo-[a]-pyrene (B[a]P), chrysene), 4-hydroxyandrostenedione (4-OHA) were obtained from Sigma-Aldrich Chemical Co. (St.Louis, MO). 2,3,7,8 TetraChloro-p-DibenzoDioxin (TCDD) was obtained from Promochem (France), ICI 182-780 (ICI) was purchased from Tocris (USA), 1,4,6-androstatrien-3,17-dione was obtained from Steraloïds (USA). Stock solutions of chemicals were prepared in dimethyl sulfoxide (DMSO) and stored at –20°C. Fresh dilutions of test chemicals were prepared before each experiment.

**Table 2:** Effective concentrations (EC_50_), maximum fold of induction measured above solvent control and relative estrogenic potencies (REP) of various compounds belonging to different chemical families.

	*Substance*	EC_50_ (nM)	*SD*	*Max. fold* *induction*	*SD*	*CV (%)*	*REP*	*n*
**Synthetic estrogens**	EE2	0.013	0.004	18	6.6	31.4	36.6	6
	HEX	0.012	0.002	24	2.1	18.6	39.1	3
	DES	0.01	0.004	22.8	1.7	36.6	45.8	3
**Natural estrogens**	E1	1.3	0.23	18.8	6.4	18.1	0.36	3
	E2	0.48	0.27	16.4	8.5	57.4	1	4
	E3	83.9	22.9	8.6	0.5	27.3	0.01	4
	4tOP	595	131.5	11.0	4.2	45.0	8.01E-04	3
**Alkylphenols**	4tPP	2541	503	10.0	3.5	19.8	1.88E-04	4
	4NPmix	406	94.4	9.3	0.3	20.9	1.17E-03	4
	4-n-NP	n.e.					-	2
	BPA	3303	933	11.5	0.5	28.3	1.44E-04	5
**bisphenol**	Zearalenone	16	3.46	20	1.0	18.5	0.030	3
**Phyto & myco-estrogens**	α-Zearalanol	>500		6.9	0.1	7.4	-	2
	α-Zearalenol	>500		5.5	1.0	9.7	-	2
	β-Zearalenol	>500		4	0.8	9.7	-	2
	Genistein	2501	6.1	8.1	0.3	0.2	1.91E-04	3
	Daidzein	n.e.					-	2
**Pesticides**	op’DDT	257	25.4	11.4	0.6	9.9	1.86E-03	3
	MXC	85	19.7	9.0	1.5	23.3	5.63E-03	3
	HPTE	477	49.2	7.4	1.6	10.3	9.99E-04	4
	Chlordecone	n.e.	-	-	-	-	-	2
	Endosulfan	n.e.	-	-	-	-	-	2
**Androgens**	Testosterone	1031	313	11.3	2.3	30	4.63E-04	3
	17α-MT	35.4		19.0	4.5		0.013	2
	11-Ketotesterone	n.e.	-	-	-	-	-	2
	DHT	2003	697	20.9	3.7	35	2.38E-04	3
	17β-trenbolone	508		13.3	4.9		9.38E-04	2
	R1881	108		8.2	0,47		-	2
**Progestagens**	Norethindrone	9.01	0.58	20.1	4.2	6.4	0.053	3
	D(−)Norgestrel	77.1	17.63	19.0	3.3	22.8	6.19E-03	2
	Progesterone	n.e						
**Other compounds**	Spironolactone	n.e						
	Dexamethasone	n.e.						
	Rifampicine	n.e.						
	Corticosterone	n.e.						
	TCDD	n.e.						
	BaP	n.e.						
	BaA	n.e.						
	diBaA	n.e.						
	Chrysène	n.e.						
	Benzophenone	n.e.						
	EtOH	n.e.						
	MetOH	n.e.						
	KMnO_4_	n.e.						

Results are expressed as mean ± standard deviation (SD).

N = number of independent experiments, n.e.: no effect, CV(%) = coefficient of variation inter-assay for EC_50_. For each experiment, 10–15 transgenic zebrafish embryos were analyzed per condition.

### Animals and Exposures to EDCs

Fertilized *cyp19a1b*-GFP transgenic zebrafish eggs were exposed to chemicals or to solvent control (DMSO; 0.01% v/v). Each experimental group consisted of 30 embryos exposed in 100 ml of water. Embryos were kept in an incubator at 28°C, under semi-static conditions. Exposures were performed from 0 dpf to 5 dpf (day post-fertilization). At the end of the exposure period, 5-dpf old zebrafish were processed for *cyp19a1b*, *gfp* expression by PCR or for fluorescence measurement by image analysis.

**Figure 3:**
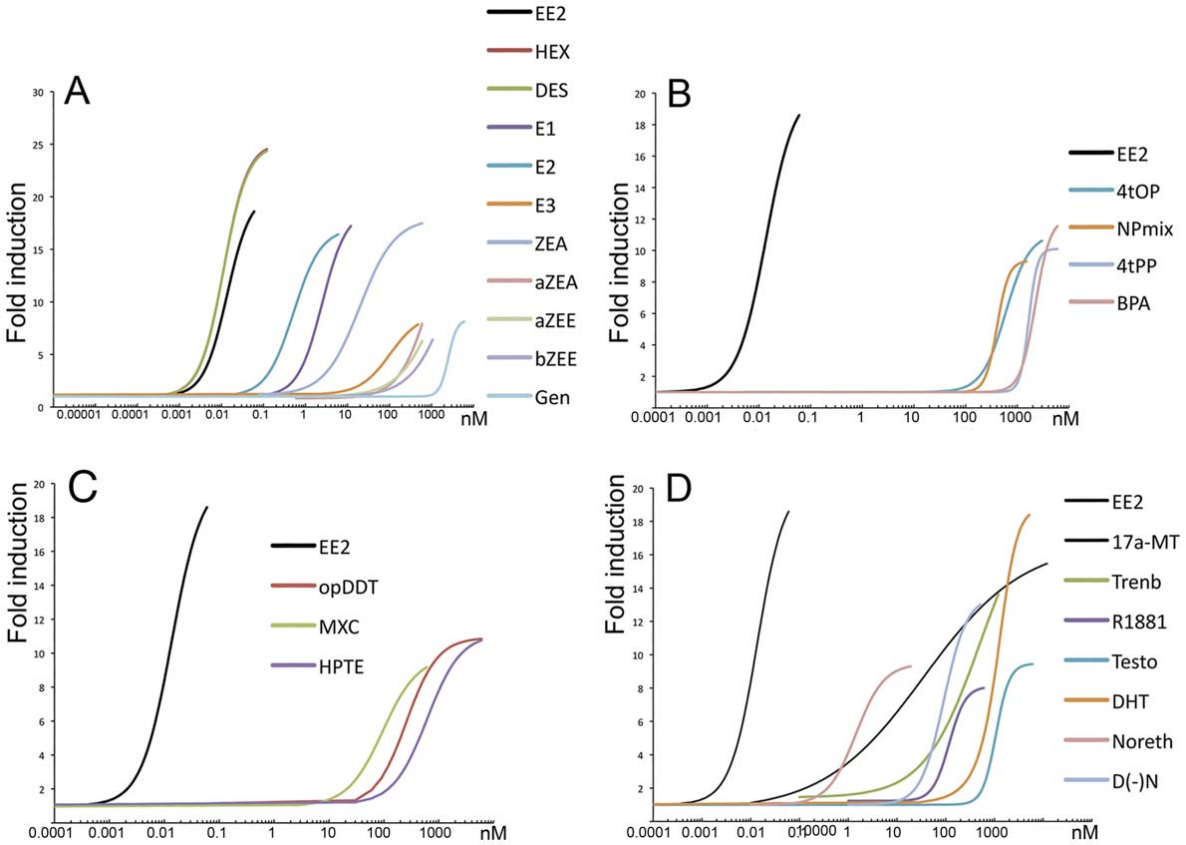
Dose-response curves of GFP induction in transgenic cyp19a1b-GFP embryos by various ligands (17α-ethinylestradiol is used as a reference). (a) Natural estrogens and pharmaceutical compounds: EE2: 17α-ethinylestradiol; E2: 17β-estradiol; E1: estrone; E3: estriol; DES: diethylstilbestrol; HEX: hexestrol; GEN: genistein; α-ZEA: α-zearalenol; α-ZEE: α-zearalanol; β-ZEE: β-zearalanol. The hexestrol curve in red is hardly visible because it is very similar to that of DES. (b) Industrial chemicals: BPA: bisphenol A; 4-t-PP: 4-t-pentylphenol; 4-t-OP, 4-t octylphenol; NPmix: mixture of nonylphenol. (c) Insecticides: o,p’DDT: 1,1,1-Trichloro-2-(2-chlorophenyl)-2-(4-chlorophenyl)ethane; MXC: methoxychlor; HPTE 2,2-bis(p-hydroxyphenyl)-1,1,1-trichloroethane. (d) Androgens: Testo: testosterone; DHT: dihydotestosterone; 17α-MT: 17α-methyltestosterone; 17β-Trenb: 17β-trenbolone; Noreth.: 17α-Ethynyl-19-nortestosterone (norethindrone); D(-)N: 13β-Ethyl-17α-ethynyl-17β-hydroxygon-4-en-3-one (levonogestrel), ICI (ICI 182-780); R1881 (metribolone): androgen receptor agonist.

For binary mixtures of estrogens, GFP induction, expressed as a percentage of response relative to E2 5 nM, was measured both for single compounds (E2, E1 and EE2) and for binary mixtures of estrogens: E1+E2 at fixed ratio of 1∶10 and E2+EE2 at fixed ratio of 1∶1. For each mixture, we performed two independent experiments. The Concentration Addition (CA) [14] and the Independent Action (IA) [15] models were used to model the theoretical concentration-response relationship for binary mixtures using a Microsoft Excel™ macro [16]. To test the compliance of experimental data with CA and IA models, residues (differences between experimental and theoretical data) were first checked for normality using Shapiro-Wilk test. Then, a Student t-test (ddl = n−2) was used to test the following H_0_ hypothesis: the mean of the residues is equal to 0 (α = 0.05). R™ (R 2.13.1, software, R development Core Team) was used for statistical analysis.

**Figure 4:**
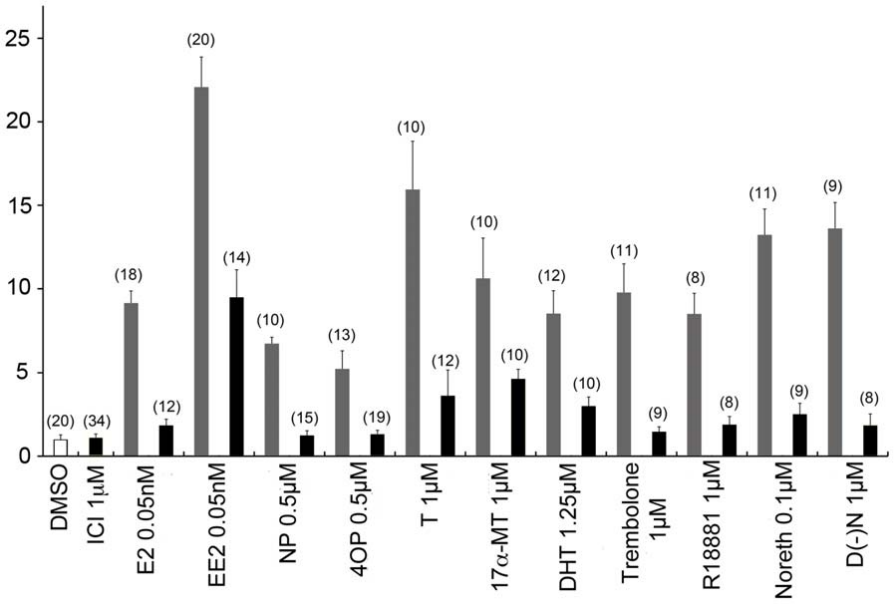
GFP expression in zebrafish embryos exposed to various ER, AR and PR ligands alone or in combination with ICI. Results are expressed as fold induction above control (means ± SEM, n = indicates the number of 5-dpf old zebrafish examined).

### RNA Extraction and Quantitative Real-time PCR

After exposure, pools of 10 zebrafish were sonicated (10 sec, three times) in 250 µL Trizol Reagent (Gibco, Carlsbad, CA, USA), and total RNA was extracted according to the manufacturer’s protocol. Reverse transcription was carried out by incubating 2 µg total RNA with 5 mM random examer oligonucleotides, 10 mM DTT, 2.5 mM dNTPs and 100 U MMLV-RT (Promega) in the appropriate buffer for 30 min at 37°C and 15 min at 42°C. Polymerase chain reaction (PCR) was performed in an iCycler hermocycler coupled to the MyiQ detector (Bio-Rad. Hercules, CA, USA) using iQ SYBR-Green Supermix (Bio-Rad) according to the manufacturer’s protocol. The following primers were used: EF-1 (fw) 5′-AGCAGCAGCTGAGGAGTGAT- 3′, EF-1 (rev) 5′-CCGCATTTGTAGATCAGATGG-3′; Cyp19a1b (fw) 5′-TCGGCACGGCGTGCAACTAC -3′, Cyp19a1b (rev) 5′- CATACCTATGCATTGCAGACC-3′; EGFP (fw) 5′-CGACGGCAACTACAAGAC -3′, EGFP (rev) 5′-TAGTTGTACTCCAGCTTGTGC -3′. Expression levels of EF-1 mRNA were used to normalize the expression of other genes. Melting curve and PCR efficiency analyses were performed to confirm correct amplification. Each experiment was performed at least twice in triplicate.

**Figure 5:**
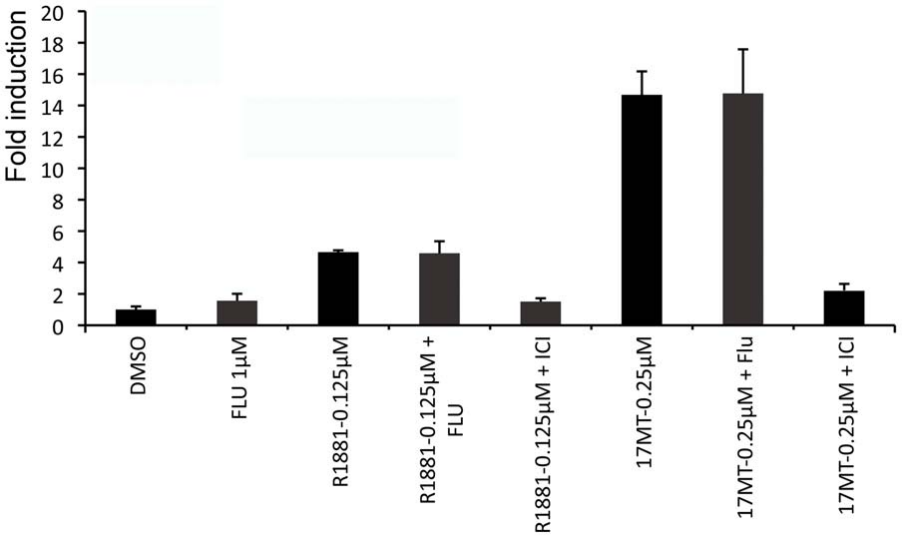
Effects of 17α-methyltestosterone and R1881 alone or in combination with either flutamide or ICI. Results are expressed as fold induction above control (means ± SEM).

### In Vivo Imaging: Confocal Microscopy

Transgenic *cyp19a1b*-GFP zebrafish were fixed in paraformaldehyde and embedded in agarose at 8 dpf. The brain was imaged with an Olympus FLUOVIEW® FV10i confocal laser scanning microscope in multiple field of view mode. The 110 images constituting each of the 9 fields of view were merged plan by plan and the resulting z-stack was reconstructed in a 3D red-green anaglyph image with the imageJ program (http://rsb.info.nih.gov/ij/).

**Figure 6:**
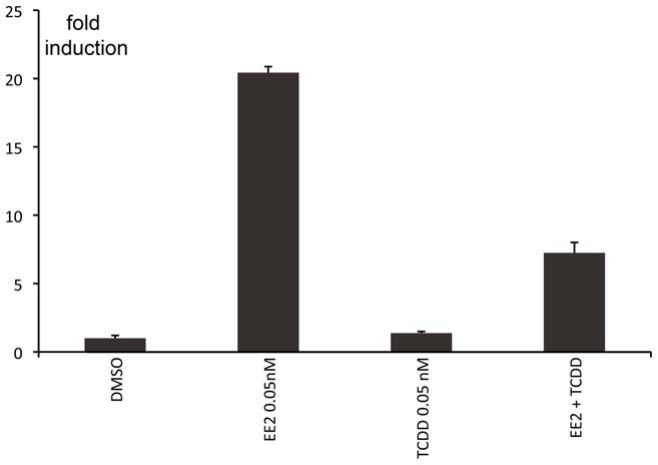
GFP expression in zebrafish embryos exposed to EE2 and TCDD (0.05 nM) alone or in combination. Results are expressed as fold induction above control (means ± SEM).

**Figure 7:**
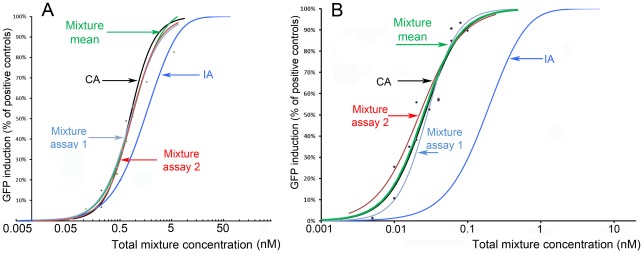
Effects of binary mixtures of estrogens on cyp19a1b-GFP expression. The combined effects of mixture of E1+E2 (ratio 1∶10) and E2+EE2 (ratio 1∶1) induced GFP expression in a concentration-dependent manner. Mixture means (green) is the mean of two independent assays, Mixture assays 1 (pale blue) and 2 (red). CA: dose response curve generated by the CA model (black). IA: dose response curve generated by the IA model (blue).

### In Vivo Imaging: Wide-field Fluorescence Microscopy

Live tg(*cyp19a1b*-GFP) embryos were observed in dorsal view and each was photographed using a Zeiss AxioImager.Z1 fluorescence microscope equipped with a AxioCam Mrm camera (Zeiss GmbH, Göttingen, Germany). All photographs were taken using the same parameters: only the head was photographed using a X10 objective, with a 134 ms exposure time and maximal intensity. Photographs were analyzed using the Axiovision Imaging software and fluorescence quantification was realized using the ImageJ software. For each picture, the integrated density was measured, i.e. the sum of the gray-values of all the pixels within the region of interest. A gray-value of 290 was defined as background value.

### Data Analysis

Chemicals were tested in at least two independent experiments. Data are expressed as a mean fold induction above solvent control ± standard error of the mean (SEM). Concentration–response curves were modelled using the Regtox 7.5 Microsoft Excel™ macro (available at http://www.normalesup.org/~vindimian/fr_index.html), which uses the Hill equation model and allows calculation of EC50. For a given chemical, EC50 was defined as the concentration inducing 50% of its maximal effect. Relative estrogenic potencies (REP) were determined as the ratio of EC_50_ of E2 to that of the test chemical. Correlation analyses between the EC_50_ of the tg(cyp19a1b-GFP) and *in vitro* assays were conducted on log- transformed EC_50_ data.

## Results

In the *cyp19a1b*-GFP zebrafish line, GFP expression, perfectly matching *cyp19a1b* expression [10], can be strongly stimulated by estradiol. As visible in Figure 1A, GFP is strictly limited to RGC of the developing brain. Figure 1B shows the distribution and organization of the RGC with a high level of resolution in the brain of an 8 days-old zebrafish larva treated with 10 nM E2. GFP-expressing RGC exhibit soma located along the brain ventricles and long cytoplasmic radial processes terminating by end-feet at the brain surface. GFP-expressing RGC can make asymmetrical divisions, generating daughter cells that undergo migration along the radial processes (Figure 1c) and rapidly loose GFP expression to gain a neuronal phenotype [7], [17].

To investigate if this model is relevant for assessing the potency of EDC to disrupt *cyp19a1b* in RGC, embryos were exposed for 5 days to increasing concentrations of 45 different compounds belonging to various chemical classes. The calculated EC_50_ based on measurements of *cyp19a1b* mRNAs, *GFP* mRNAs or *in vivo* imaging showed that the 3 methods yielded similar results (Table 1), indicating that GFP expression reflects the response of the endogenous gene. Figure 2 shows examples of the GFP signal generated by different active compounds. In 5 days-old controls, GFP expression is weakly detectable in the preoptic area, while embryos exposed to active compounds exhibit a stronger fluorescence signal with a much wider distribution from the anterior telencephalon to the caudal hypothalamus.

Selected compounds with well-known estrogenic activity included pharmaceuticals estrogens, natural estrogens, phyto and myco-estrogens, and environmental chemicals. Most of them were able to induce GFP expression in a concentration-dependent manner, but clear differences in terms of both EC_50_ and maximal induction were observed (Table 2, Figures 2 and 3). As expected, the synthetic estrogens EE2, HEX and DES were the most active compounds (Figure 3*a*) characterized by extremely low EC_50s_ (10 pM) and maximal inductions around 20 times the basal expression in controls. Based on their REP (relative estrogenic potency), they are much more potent than E2 by a factor 37–46. The natural estrogens, E2 and E1 yielded very similar responses (Figure 3*a*), while E3 was active at much higher concentrations with an REP 175-fold lower than E2. The myco-estrogen zearalenone (Zea) and its metabolites α-Zea, α-Zee and β-Zee exhibited different response patterns (Figure 3*a*). Zea induced a full concentration-dependent response curve similar to those of the E2 and E1, while the three Zea metabolites did not elicited complete concentration-response curves. Among isoflavones, genistein was the only active compound, though at relatively high concentrations, and daidzein was inactive.

Among the various industrial chemicals tested, all alkylphenolic compounds were active, yielding similar concentration-dependent responses with the exception of the linear alkylphenol 4-n-NP that was inactive (Figure 3*b*). NPmix and 4-*tert*-OP exhibited similar estrogenic potencies while 4-*tert*-PP was 6-fold less active than NPmix. In comparison, the NP mixture was 8-fold more active than BPA. Several organochlorine pesticides were also tested (Figure 3*b*). The DDT-related compounds, o,p’-DDT and MXC, induced strong GFP expression with similar response patterns (Figure 3*c*). Endosulfan and chlordecone were inactive. The strong effect of MXC is of interest since it is known that its estrogenic potency is due to biotransformation into estrogenic metabolites. Among them, bis-desmethyl-MXC (HPTE) was capable of inducing GFP expression in RGCs. Interestingly, the EC50 of HPTE was higher than the EC50 for MXC, which could reflect the additive effect of several estrogenic metabolites derived from MXC and/or a higher excretion rate of HPTE compared to MXC. Again, this demonstrates the xenobiotic biotransformation capacities of embryos.

This is further illustrated by the fact that several natural and synthetic androgens also induced GFP expression. This was the case of the aromatizable androgens, T and 17α-MT (Figure 3d) an effect due to aromatization since it is blocked by co-exposure with the ER antagonist ICI 182,780 (Figure 4). In agreement, confirming previous studies [18], the non-aromatizable androgen 11-KT was totally inactive, while DHT, a non-aromatizable androgen, strongly up-regulates GFP expression (Figure 3d), an effect blocked by ICI 182-780 (Figure 4). Based on their REP, the non-aromatizable synthetic androgens, 17β-trenbolone and R1881, were 4200 and 1000-fold less active than E2, but induced strong GFP expression by a factor of 20 and 13, respectively (Figure 3d). These effects could not be blocked by the androgen receptor antagonist flutamide (Figure 5), but were in contrast blocked by co-exposure with ICI (Figure 4) In addition, two synthetic progestins, norethindrone and levonorgestrel, commonly used in oral contraception and post-menopausal disorders, induced GFP expression in a concentration-dependent manner (Figure 3d), while progesterone was inactive. Based on their respective EC_50_, norethindrone was 8.5-fold more active compared to levonorgestrel and both exhibited lower estrogenic potencies compared to E2 (Table 1). Inhibition of progestins-induced fluorescence in embryos co-exposed with ICI revealed the involvement of ERs in mediating this effect (Figure 4). To further evaluate the specificity of the assay in detecting estrogenic activity, several other compounds were selected. The GR agonist dexamethasone, the MR antagonist spironolactone, the PXR agonist rifampicine, several aromatase inhibitors such as anastrosole, androstatrienedione and 4-hydroxyandrostenedione, the UV-filter benzophenone, ethanol, methanol and potassium permanganate were all unable to induce GFP expression.

Because of the ubiquitous character of dioxin-like compounds as environmental contaminants, agonists of the aryl hydrocarbon receptor (AhR) were evaluated (TCDD, BaP, BaA, diBaA, chrysene). None of the AhR agonist ligands were able to induce GFP expression in RGCs in agreement with previous data [19]. However, co-exposure of embryos to TCDD and EE2, significantly down-regulated the EE2-induced fluorescence confirming the anti-estrogenic effect of TCDD on ER-mediated mechanisms (Figure 6).

Combination effects of binary mixtures of estrogens were assessed using the fixed-ratio method. Experimental designs of mixtures were optimized so that the mixture concentrations covered a large range of effect predicted by the CA model. The combined effects of mixture of E1+E2 (ratio 1∶10) and E2+EE2 (ratio 1∶1), induced GFP expression in a concentration-dependent manner which were predicted by CA model [14] but not by IA model [15] (Figure 7).

## Discussion

This study confirms the high sensitivity of the *cyp19a1b* gene to estrogens and xeno-estrogens in the RGC context [4], [6], [18]. The tg(*cyp19a1b*-GFP) embryo assay is sensitive, fast, and cost effective for estrogen mimic screening. Twenty-one out of the 45 compounds tested induced GFP expression in a concentration-dependent manner through ER binding. For several of them, this study is the first to report estrogenic activity in vivo. In addition, this study demonstrates that a wide range of EDC targets RGC in fish brain, raising concern about the consequences of their actions on brain development and functioning.

The synthetic estrogens (EE2, DEX, HEX) were 37 to 49 times more potent than E2 with EC_50s_ similar to those previously reported in the most sensitive fish and human cell-based *in vitro* assays [8], [20], [21]. In transgenic zebrafish stably expressing ERE-Luciferase [21], EC_50s_ for EE2 and E2 were 10 and 20 times higher, respectively, than those reported using the tg(*cyp19a1b*-GFP) further illustrating the sensitivity of the cyp19a1b gene to synthetic estrogen and the sensitivity of this line. Zearalenone and zearalenone metabolites have been well described as ER agonists in both fish and human *in vitro* systems [22]. In this study, zearalenone exhibited a strong concentration-dependant induction of GFP while zearalenone metabolites induced partial concentration-response, indicating that zearalenone metabolites generally behave as partial agonists of fish ERs [20], [23]. In agreement, zearalenone exhibited a comparably strong *in vivo* effect on reproduction, notably vitellogenin induction zebrafish, despite its low *in vitro* estrogenic potency [24]. The phyto-estrogen genistein clearly stimulated GFP expression in RGCs in agreement with previous data [25]. Interestingly, in tg(5xERE:GFP) fish genistein induced fluorescence in heart and liver, but not in brain [26].

In this assay, industrial chemicals with known estrogenic activity, such as alkyphenolic compounds (4NPmix, 4-t-OP, 4-t-PP), BPA, o,p’DDT, MXC, and its estrogenic metabolite HPTE, were active, in contrast with the fact that NP had no effect in ERE-luc zebrafish [21], *vtg*-GFP [27] and 5xERE:GFP [26]. Differences were also noticed regarding the effect of BPA. In 5xERE:GFP larvae, BPA activates ER transcriptional activation only in heart and liver [26], whereas BPA induces GFP expression in RGCs of developing tg(*cyp19a1b*-GFP) further confirming recent data (15) of BPA on cyp19a1b expression in wild type zebrafish. Importantly, in mammals BPA adversely affects brain development and brain sexual differentiation [28], [29].

In addition to the extreme sensitivity of the *cyp19a1b* gene, the biotransformation capacity of the tg(*cyp19a1b*-GFP) embryo is a clear advantage over *in vitro* assays. This is exemplified by MXC whose metabolites OH-MXC and HPTE directly interact with ER and potentially show long lasting additive effects [30]. Testosterone and 17a-MT, and the non-aromatisable DHT, but not 11-KT, were able to induce *cyp19a1b* expression in RGCs in an ER-dependant manner. While aromatase converts androgens into estrogens that subsequently bind to ERs to activate the *cyp19a1b* promoter [4], [6], [18], DHT effect involves conversion into 5α-androstane-3β,17β-diol, a metabolite of DHT with known estrogenic activity. Conversion of DHT into diols requires 5a-reductase and 3b-hydroxysteroid dehydrogenase, both of which are expressed in the brain of developing fish [31] and rodents [32].

17β-trenbolone acetate is a potent androgen extensively used in the United States as a growth promoter in beef. It is a recognized reproductive toxicant in fish [33]. R1881 is the 17-methylated derivative of 17β-trenbolone and is also a potent non-aromatizable androgen agonist of fish and human AR [34]. To our knowledge, this is the first report on the capacity of 17β-trenbolone and metribolone to activate an ER-dependent gene in a vertebrate. The metabolic pattern of 17β-trenbolone acetate revealed the presence of two major metabolites, 17α-trenbolone and trendione that have low affinity for androgen receptor as compared to 17β-trenbolone acetate [35], however their affinity towards ERs is unknown [36]. Progesterone and 19-Nor-testosterone derivatives, used in contraception, behaved differently in tg(*cyp19a1b*-GFP) embryos. Progesterone had no activity as expected from its lack of estrogenicity [27], [37]. But, we show for the first time that norethindrone and levonorgestrel, both of which are present in surface waters [38], were very active. In mammals, none of these compounds binds ERs, but they elicit estrogenic effects when they are metabolized into 3β, 5α-tetrahydro norethindrone or norgestrel derivatives, which are likely responsible for the observed *in vivo* estrogenic effects of the parent compounds [39], [40].

We also addressed the question of the combination effects of mixture of estrogenic hormones. We show that mixture of E2 and EE2 (E2+EE2; 1∶1) as well as mixture of E1 and E2 (E1+E2; 1∶10) acted in an additive manner on *cyp19a1b*-driven GFP expression that was predicted by the CA model, in agreement with previous data on vitellogenin synthesis [41] or on zebrafish *cyp19a1b*-luciferase activity in vitro [8]. It highlights the interest of the tg(cyp19a1b-GFP) in combination with CA models to assess combined effect of estrogenic compounds.

In conclusion, the tg(c*yp19a1b*-GFP) line clearly emerges as a simple, fast and reliable *in vivo* assay for monitoring the capacity of any chemical or its metabolites to activate ER-signalling in vivo at very early critical developmental stages. It is based on the use of an endogenous promoter and thus shows of a true physiological brain-specific response. Its sensitivity is outstanding and comparable to the most performing *in vitro* assays [42]. In complement of the *in vitro* assay using the same *cyp19a1b* promoter [8], this in vivo assay will permit taking into account the biodisponility and pharmaco-dynamics of chemicals. This will enhance the efficiency and accuracy of EDCs testing strategies while meeting the 3R policy (replacement, reduction, refinement) that is enforced by the OECD (Organisation for Economic Co-operation and Development) and the main environmental agencies worldwide [43].

Finally, although the potential consequences of such exposures are unknown, the present data showing direct effects of EDCs on gene expression in radial glial progenitors raise several serious issues in the context of risk assessment. One of them is to evaluate to what extent the present findings may apply to other vertebrates. Some studies indicate that estrogens indeed affect early brain development in rodents [44], [45], [46], [47], [48], but there is a lack on data the expression on steroidogenic enzymes, notably aromatase, and estrogen receptors, notably ERb in the developing brain. Similarly, the roles of steroids in early aromatase expression [49] are unknown. Additionally, the potential production and effects of beta-diol, sometimes referred to as the “second estrogen”, have just started to receive some attention [50], albeit the present work recalls that this alternative pathway should not be forgotten in the context of developing animals.
